# Longitudinal Lung Function Decrease in Subjects with Spontaneous Healed Pulmonary Tuberculosis

**DOI:** 10.1371/journal.pone.0164039

**Published:** 2016-10-05

**Authors:** Seung Heon Lee, Amy M. Kwon, Hae-Chung Yang, Seung Ku Lee, Young Kim, Jong Hyun Choi, Je Hyeong Kim, Chol Shin

**Affiliations:** 1 Division of Pulmonary, Sleep, and Critical Care Medicine, Department of Internal Medicine, Korea University Ansan Hospital, Ansan, Republic of Korea; 2 Institute of Human Genomic Study, Korea University Ansan Hospital, Ansan, Republic of Korea; Fundació Institut d’Investigació en Ciències de la Salut Germans Trias i Pujol, Universitat Autònoma de Barcelona, SPAIN

## Abstract

**Objective:**

We compared the longitudinal course of post-bronchodilator Forced Expiratory Volume in 1 second (pFEV1) over a 10-year period in subjects with spontaneous healed pulmonary tuberculosis (SHPTB) with that in normal subjects.

**Methods:**

We prospectively investigated 339 subjects with SHPTB and 3211 normal subjects. pFEV1 values measured biannually over 10 years were analyzed using mixed effects model.

**Results:**

At baseline, there were no differences in gender, smoking amount, and mean height, except mean age (50.0 ± 8.1 VS. 48.1 ± 7.3, *P*< 0.001) between the SHPTB and normal group. 52% of the 339 participants with SHPTB and 56% of the 3211 normal participants participated till the end of study. According to the final model, the SHPTB group showed significantly larger decrease in the average pFEV1 over the time than the normal group (P< 0.001) adjusted for gender, age, height, smoking pack years, and time effects. Especially, the interaction effect between time and group was statistically significant (P = 0.036).

**Conclusion:**

The average lung function in terms of pFEV1 decreases faster in subjects with SHPTB than in normal individuals over time.

## Introduction

There has been debate over the course of lung function in human life. The forced expiratory volume in one second (FEV1) is the most widely used metric for lung function in clinical practice and research. It is recognized that during the childhood and adolescence lung function naturally increases, reaching a plateau at 20–25 years of age [1]. After the plateau phase, the FEV1 begins to decrease, and decline can be accelerated by aging, airways hyper-responsiveness, childhood infections, air pollution, occupational hazards [2], and underlying chronic obstructive pulmonary disease [3].

Moreover, pulmonary TB following treatment can cause pulmonary function impairment. Some reports suggest that TB can cause subsequent airflow obstruction with decline of FEV1 and FEV1/FVC ratio [4,5], restrictive lung disease with decline of FVC, or mixed lung disease with both [4,6]. Furthermore, it seems that the degree of obstructive airway changes in patients treated for TB increases with the number of cigarettes smoked, age, and the extent of the initial TB [7]. Additionally, it was reported in a cross-sectional study that pulmonary function is different even between the patients with active TB and latent tuberculosis infection (LTBI) [8]. However, considering South Korea’s intermediate TB burden (an incidence rate in 2010 of 97/100000) [9], pulmonary function impairment even in subjects with spontaneous healed TB lesion which can be candidates for LTBI treatment cannot be overlooked.

The spontaneous healed TB, when viewed on the CXR, can show non-calcified nodules with distinct margins, calcified nodules, discrete linear or reticular fibrotic scars with or without calcification, pleural thickening or calcification, focal bronchiectasis, pericicatrical emphysema, and lymph node calcification without clinical symptoms [10,11]. It is plausible that the inflammation results in a structural remodeling of the airway wall, with increasing collagen content and scar tissue formation, that narrows the lumen and produces fixed airway obstruction manifesting clinically as chronic obstructive pulmonary disease (COPD) [12].

No data based on longitudinal follow up are available for the effects of spontaneous healed TB lesion on lung function. Therefore, there is an urgent need for a study that is constructed in a longitudinal fashion, looking at decline over time to compensate for the over- [13] or under-estimation [14] of actual decline of pulmonary function on cross sectional analysis. The aims of this study was to compare the longitudinal decline of pFEV1 over a 10-year period between subjects with CXR-detected spontaneous healed pulmonary tuberculosis (SHPTB) and normal subjects.

## Methods

### Study Cohort and study design

This study is based on the Korean Genome and Epidemiology Study (KoGES). The KoGES, which started in 2001, is an ongoing population-based cohort study of Korean adults aged 40–69 years. The participants in this study were residents of Ansan, an industrialized city of 700000 people located about 32 km south-west of Seoul. From 2003–2004, those enrolled in our study participated in baseline physical examination, blood pressure, pre- and post-bronchodilator spirometry, electrocardiography and blood tests, and answered a questionnaire designed to collect information including respiratory symptoms, smoking history, occupation, coexisting medical conditions as well as basic information. Participants were followed biannually with same repeated tests including post-bronchodilator spirometry. We examined the accumulated pulmonary function data of this cohort for 10 years, including the accompanying biannually collected information. For this pulmonary function study, we excluded the subjects with confirmed asthma, COPD, or baseline FEV1/FVC<70 at the time of enrollment. Also, for the purpose of estimating the change of post-bronchodilator FEV1 in normal subjects and subjects with spontaneous healed tuberculosis with scar lesions detectable on CXR, we excluded the subjects who were taking medication for airway disease at the time of enrollment or during follow up. Moreover, for initial enrollment, we excluded the subjects with a history of TB or TB medication, to exclude the effects of destructive lung changes caused by active pulmonary TB. Also, we excluded the subjects who developed active TB during the 10 years of follow up for accurate analysis. The procedures were performed in accordance with institutional guidelines, and were approved by an institutional review board of the Korea University Ansan Hospital (IRB approval number: AS0624). Written Informed consent was obtained from all study participants.

### Definition of SHPTB and normal subjects in chest radiograph

SHPTB was defined as presence on CXR of non-calcified nodules with distinct margins, calcified nodules, discrete linear or reticular fibrotic scars with or without calcification, or pleural thickening or calcification without clinical symptoms [10,11]. But, destructive change on CXRs were excluded from this groups. For SHPTB group and normal group, the subjects with a history of pulmonary TB or evidence of past or present TB were all excluded. CXRs were reviewed by both a chest radiologist and a pulmonary specialist. If there was a discrepancy in the reading of the CXRs by the primary reader, the final interpretation was reached by consensus.

### Spirometry for pulmonary function test

Spirometry was performed by three well-trained pulmonary technicians following the 1994 ATS recommendations [15], using a spirometer(Vmax-229, Sensor-Medics, Yorba Linda, CA, USA) for all subjects. The predicted forced expiratory volume in one second (FEV1) and forced vital capacity (FVC) were measured using the method of Morris [16]; the patients performed forced expiratory maneuvers until three measurements met the ATS guideline recommendations. The subjects performed a maximum of eight forced expirations; those who were unable to perform three expiratory maneuvers that met ATS guidelines were excluded. Two doses of fenoterol hydrobromide (Berotec^®^, Boehringer Ingelheim, Ingelheim, Germany) 200 μg were administered 1–2 min apart. The forced expirations were repeated 15 min after administration of the bronchodilator.

### Statistical analysis

The purpose of this study was to examine the difference of changes in pFEV1 between SHPTB and normal subjects during a 10 year- follow up period. A mixed effect model was used to estimate longitudinal course of pFEV1 as well as the rate of change over time in pFEV1 for the two groups using all available data. Mixed effect models are useful with longitudinal data because the models take into account correlation structure among repeated measurements within a subject. According to log-likelihood values, the best fit of correlation structure was determined as compound symmetry structure, and random effects were applied to slope as well as intercept. The shape of curves was assumed to be linear, and interaction between group and time was added to the model. Potential confounding factors (age, gender, pack-years, height) and their interactions were tested under α = 0.05, and the model was adjusted for significant covariates.

Baseline characteristics of the participants were summarized using descriptive statistics in Table 1. Continuous variables were summarized with mean and standard deviation, and discrete variables were summarized with the number of counts and percentages. All analyses were conducted using SAS 9.3 (NC, USA).

**Table 1:** Characteristics of the participants with SHPTB and comparison normal subjects at baseline.

Characteristics	SHPTB (n = 339)	Normal subjects (n = 3211)	P value
Age (yr)	50.0 ± 8.1	48.1 ± 7.3	<0.001
Male (%)	182 (53.7%)	1674 (52.1%)	0.58
Current smoker	68 (20.1%)	763 (23.9%)	0.130
Smoking Pack years	7.6 ± 14.2	7.9 ± 13.1	0.23
Height (cm)	160.7 ± 8.2	161.3 ± 8.5	0.32
pFEV1 (ml)	3001.48 ± 637.66	3107.74 ± 645.61	0.004
pFEV1 percent of predicted value	112.76 ± 15.25	113.25 ± 14.08	0.66
FEV1/FVC (%)	83.18 ± 5.78	82.77 ± 5.15	0.12

SHPTB = spontaneous healed pulmonary tuberculosis; pFEV1 = Post-bronchodilator forced expiratory volume in one second; FVC = Forced vital capacity.

## Results

### Participant characteristics at baseline

**Fig 1:**
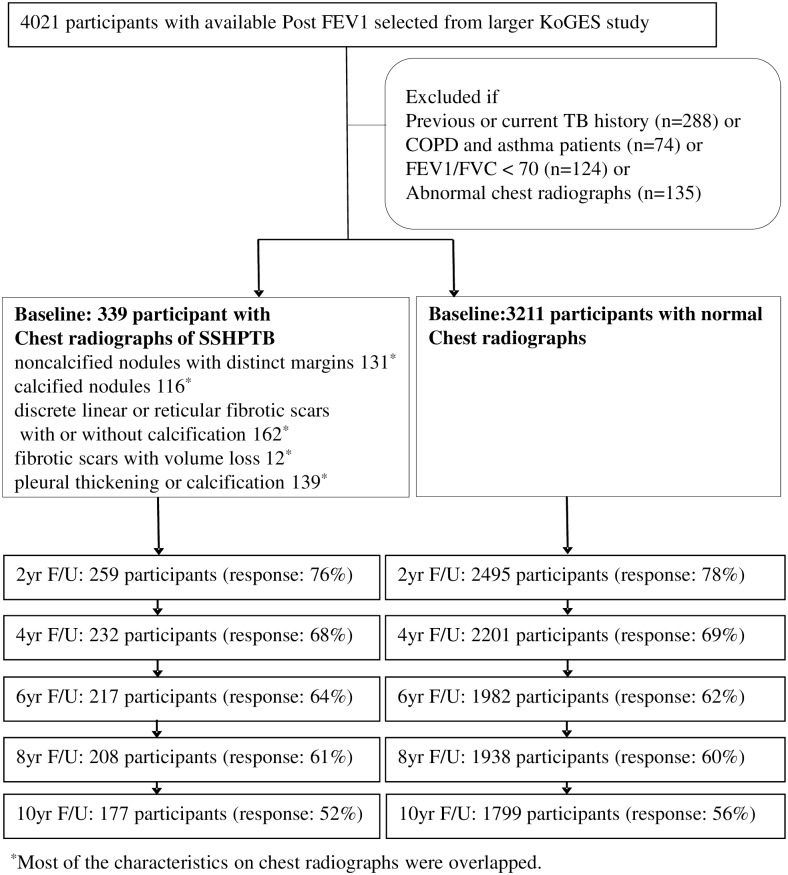
Participants flowchart for ten years of follow up. FEV1 = forced expiratory volume in 1 second; yr = year; FU = follow-up.

A total of 3550 participants were included in this Cohort Study (Table 1). Pulmonary functions including pFEV1s were measured biannually over 10 years, and 56% of the 3211 normal participants and 52% of the 339 participants with SHPTB were assessed until the end of study. Of the 3221 normal participants and 339 participants with SHPTB, 1779 and 177 had five FEV1 assessments, 1938 and 208 had four, 1982 and 217 had three, 2201 and 232 had two, 2495 and 259 had only one, respectively (Fig 1). The baseline characteristics of the participants are shown in Table 1, categorized by SHPTB and normal group.

The group had 53.7% and 52.1% males, respectively, without statistical difference (*P* = 0.58). The mean age was higher in SHPTB group than in normal group (50.0 ± 8.1 years and 48.1 ± 7.3 years, *P*< 0.001). The percentage of current smoker at baseline were 20.1% and 23.9%, with an average smoking pack years of 7.6 ± 14.2 and 7.9 ± 13.1, respectively (*P* = 0.23). The mean height were 160.7 ± 8.2 cm and 161.3 ± 8.5 cm, respectively (*P* = 0.32). The mean pFEV1 were significantly lower in the SHPTB group (3001.48 ± 637.66 ml) than in the normal group (3107.74 ± 645.61 ml) (*P* = 0.004), but the mean pFEV1% predicted of the study samples were similar as 112.76 ± 15.25% and 113.25 ± 14.08%, respectively (*P* = 0.66). The mean pFEV1/FVC of the two groups were 83.18 ± 5.78% and 82.77 ± 5.15% (*P* = 0.12).

### Pulmonary functions between SHPTB and Normal subjects

Outcomes from the mixed effects models are presented in Table 2. Declining slopes ranged as steep as -24.030ml (*P*< 0.001) per additional age, +38.231ml (*P*< 0.001) per additional height (cm), and -2.425ml (*P*< 0.001) per additional pack · year for pFEV1. As significant covariates, gender (P<0.001), age (P<0.001), height (P<0.001), smoking pack years (P<0.001), time (P<0.001) and the interaction between time and group (P = 0.036) were selected, and their confounding effects were adjusted in the model. With adjustment of those covariates, the SHPTB group showed significantly lower pFEV1 than the normal group (P = 0.011). Especially, pFEV1 was estimated lower in male than female.

**Table 2:** Fixed effects from mixed effects models estimating the longitudinal course of post FEV1 during 10 years between SHAPTB group and normal group.

Variables	pFEV1 (mL) Estimate	SE	P value^a^
Intercept	-1615.770	190.329	0.000
Gender (Male)	-434.885	18.629	0.000
Age	-24.030	0.773	0.000
Height	38.231	1.065	0.000
Smoking Pack Years	-2.425	0.386	0.000
Group (SHPTB)	-48.842	19.093	0.011
Time	-33.806	7.611	0.000
Group (SHPTB) x Time	-2.656	1.267	0.036

pFEV1 = Post-bronchodilator forced expiratory volume in one second; SE = standard error; SHPTB = spontaneous healed pulmonary tuberculosis; CXR = chest radiograph.

^a^Adjusted for age, sex, height, smoking amount, and time interaction with each variables.

### Decrease in pFEV1 between SHPTB group and normal group

**Fig 2:**
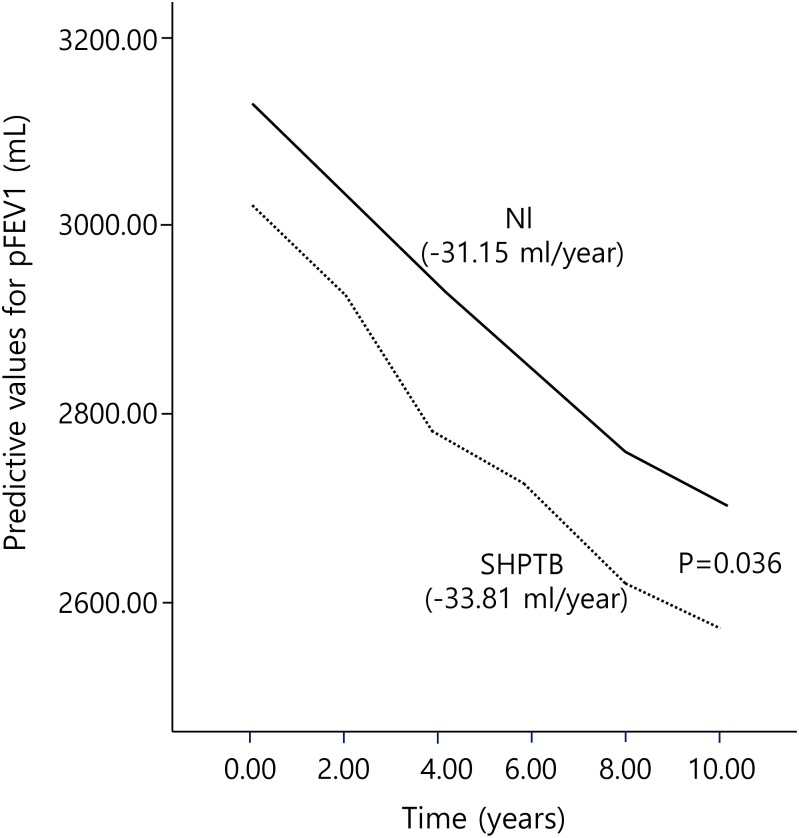
Longitudinal course of post FEV1 from mixed effects model for 10 years in the normal and SHPTB groups. Nl = normal group; SHPTB = spontaneous healed pulmonary tuberculosis group.

Fig 2 shows the longitudinal course of pFEV1 for the SHPTB group and normal groups estimated by the mixed effects model. As presented in Table 2, decrease in pFEV1 of SHPTB group was faster than normal group as time went on (pFEV1 estimate = -2.656, *P* = 0.036). The rate of pFEV1 decrease in the SHPTB group was 33.81ml/yr and the rate of that in the normal group was 31.15ml/yr, respectively.

## Discussion

Our study showed substantial faster decrease of the pFEV1 over time in individuals with SHPTB than in normal individuals, even though pFEV1 is supposed to be lower in individuals with SHPTB than in normal individuals at cross-section time.

The elderly population with SHPTB in South Korea has higher prevalence of active pulmonary TB development, because TB was highly prevalent during the youth of most persons born during 1955–1965 after the Korean War [17]. Therefore, we adjusted the age that can have an effect on FEV1 decline, as well as smoking [18], height, and sex [2,19]. The observed faster decrease of the pFEV1 in SHPTB group can be attributed to airway obstruction developed by TB involvement in the endobronchial trees. Sub-mucosal TB involvement with nonspecific inflammation, bronchiectatic change with bronchial tree obstruction, and emphysematous change with fibrotic band supposed to cause airway obstruction in pulmonary function [7,20].

Our study suggest longitudinal declines of pFEV1 in subjects with SHPTB on CXR manifestation in an intermediate TB burden country like South Korea; in our study we excluded all the subjects who have the history of TB medication or TB treatment, although latent TB infection was not confirmed by diagnostic tool such as tuberculin skin test (TST) or interferon gamma release assay (IGRA). In the same context, further investigation for LTBI treatment effect against pFEV1 decline over time for the immunocompromised subjects with SHPTB can be investigated to elucidate the mechanism of obstructive airway disease in SHPTB.

The prevalence of obstructive airway disease defined by FEV1/FVC < 70 [21] for consecutive year was significantly different between SHPTB group and normal group [4/339 (1.2%) VS. 65/3211 (0.2%), *P* = 0.01]. The difference in the rate of pFEV1 decrease over 10 years was only 26.6cc, which is comparable to the amount of the average yearly decline from 25 years of age in adult [2]. Moreover, SHPTB group showed lower pFEV1 by -48.842ml in comparison with normal group on average during the study period for 10 years, and decreasing rate of pFEV1 was faster in SHPTB group (33.82ml/yr) than in normal group (31.15ml/yr) additionally. Therefore, effort on prevention and TB control with smoking cessation in countries with intermediate TB burden supposed to reduce the prevalence rate of obstructive airway disease.

However, there was no difference in the rate of post-bronchodilator FVC (pFVC) decrease adjusted by sex, age, height, and smoking between SHPTB group and normal group (P = 0.46). The reason for this seems to be the relative healing change in SHPTB group, as visualized by CXRs, in contrast to the destructive change resulting in restrictive change of pulmonary function after advanced active pulmonary TB [8].

There are some limitations to our study. First, we cannot assure that the subjects with SHPTB are all the subjects with LTBI only based on CXRs without TST or IGRA. Secondly, we could not conduct chest computerized Tomogram (CT) or bronchoscopy for all participants to exclude other possible lesion, such as anthracofibrosis, as a cause of airway obstruction. Thirdly, there can be cohort effects by environmental and nutritional changes, and period effects by technical experience for pulmonary function fulfillment. Lastly, the number of the subjects with SHPTB until last follow up was relatively smaller than that of normal subjects, and the concomitant clinical data of the enrolled participants such as dyspnea scale was not analyzed.

Despite the limitations, we can conclude that the pFEV1 decreases faster in individuals with SHPTB on CXRs than in normal individuals over time, with a total difference of 26.6 ml on pulmonary function test over 10 years. Therefore, appropriate TB control with prevention is important as smoking cessation to reduce the decline of FEV1 over time.

## Supporting Information

S1 Fig A chest radiograph representing fibrotic scar with volume loss. (TIF)

S2 Fig A chest radiograph representing non- and calcified-nodules with distinct margins. (TIF)

S3 Fig A chest radiograph representing non- and calcified-nodules with discrete linear lesions. (TIF)

S4 Fig A chest radiograph representing non-calcified nodule with discrete linear scar, volume loss, and pleural calcification. (TIF)

S5 Fig A chest radiograph representing pleural thickening. (TIF)

S6 Fig A chest radiograph representing small calcified nodules. (TIF)

S7 Fig A chest radiograph representing non-calcified nodules, calcified nodules, discrete linear fibrosis, volume loss, and pleural thickening all together. (TIF)
