# Supplementary figures and images for: Longitudinal Lung Function Decrease in Subjects with Spontaneous Healed Pulmonary Tuberculosis
Source: PLoS One. 2016 Oct 5;11(10):e0164039. doi: 10.1371/journal.pone.0164039 (PMC5051937; doi:10.1371/journal.pone.0164039)

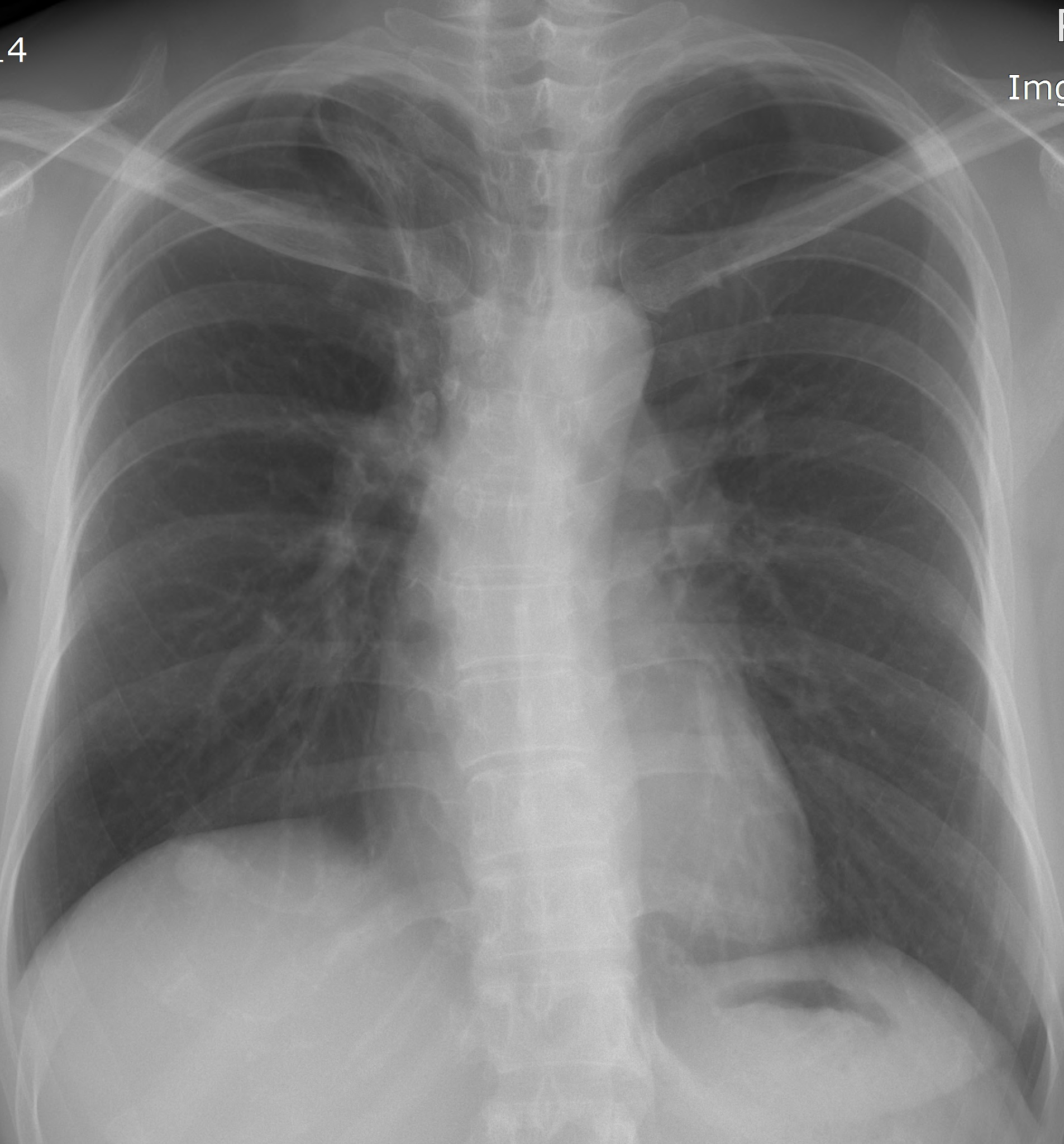

Supplement: S1 Fig — (TIF) [file pone.0164039.s001.tif]

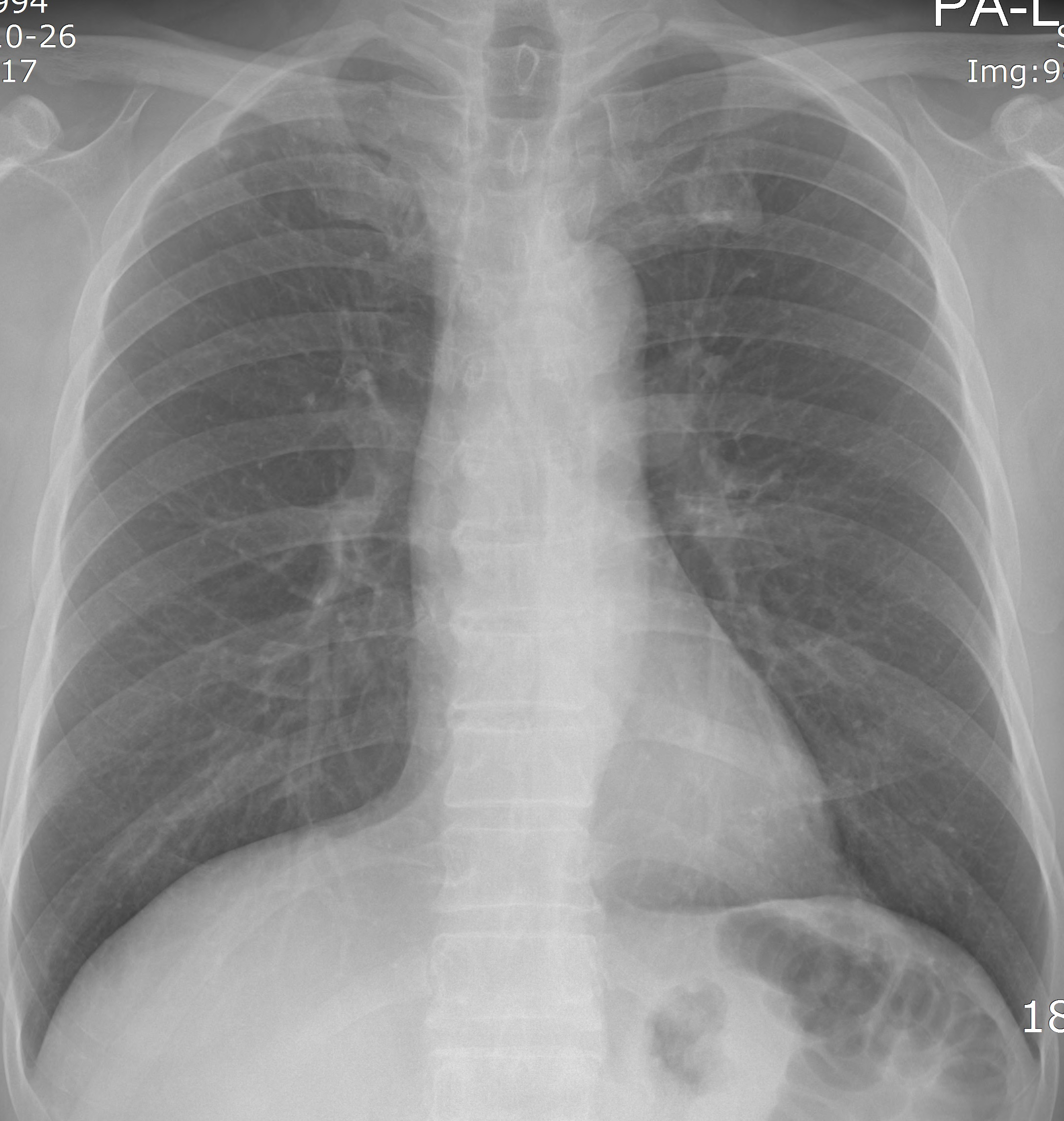

Supplement: S2 Fig — (TIF) [file pone.0164039.s002.tif]

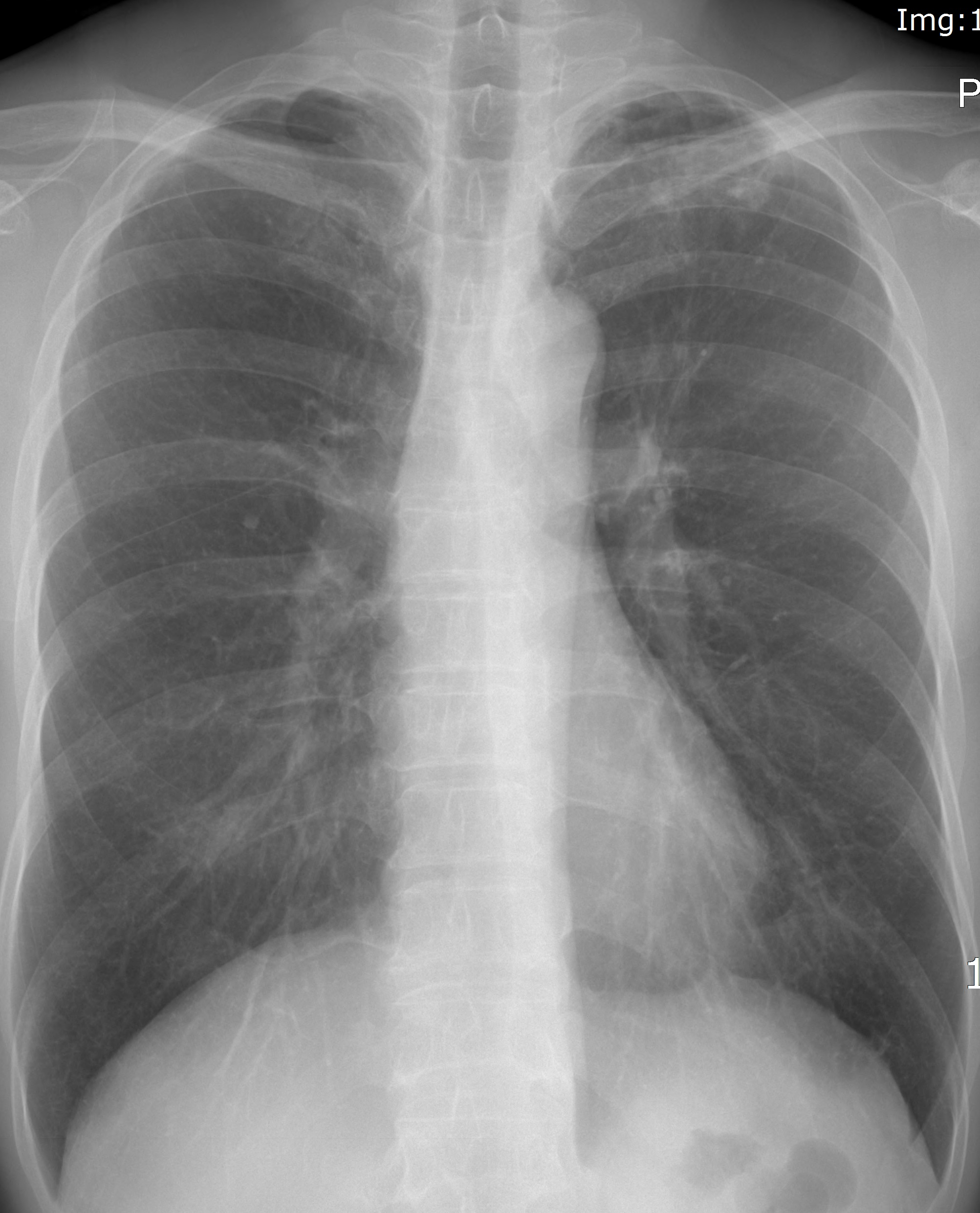

Supplement: S3 Fig — (TIF) [file pone.0164039.s003.tif]

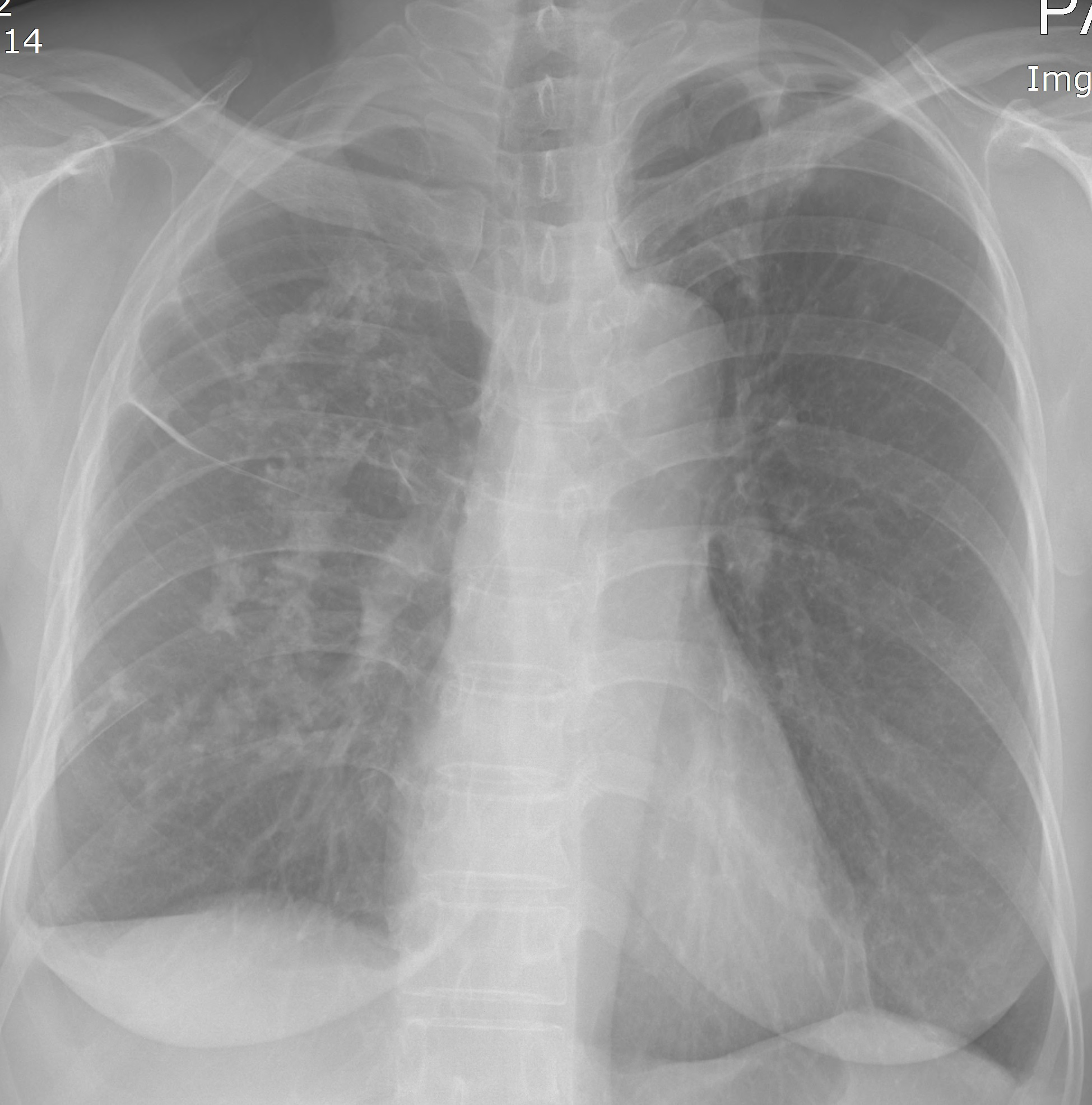

Supplement: S4 Fig — (TIF) [file pone.0164039.s004.tif]

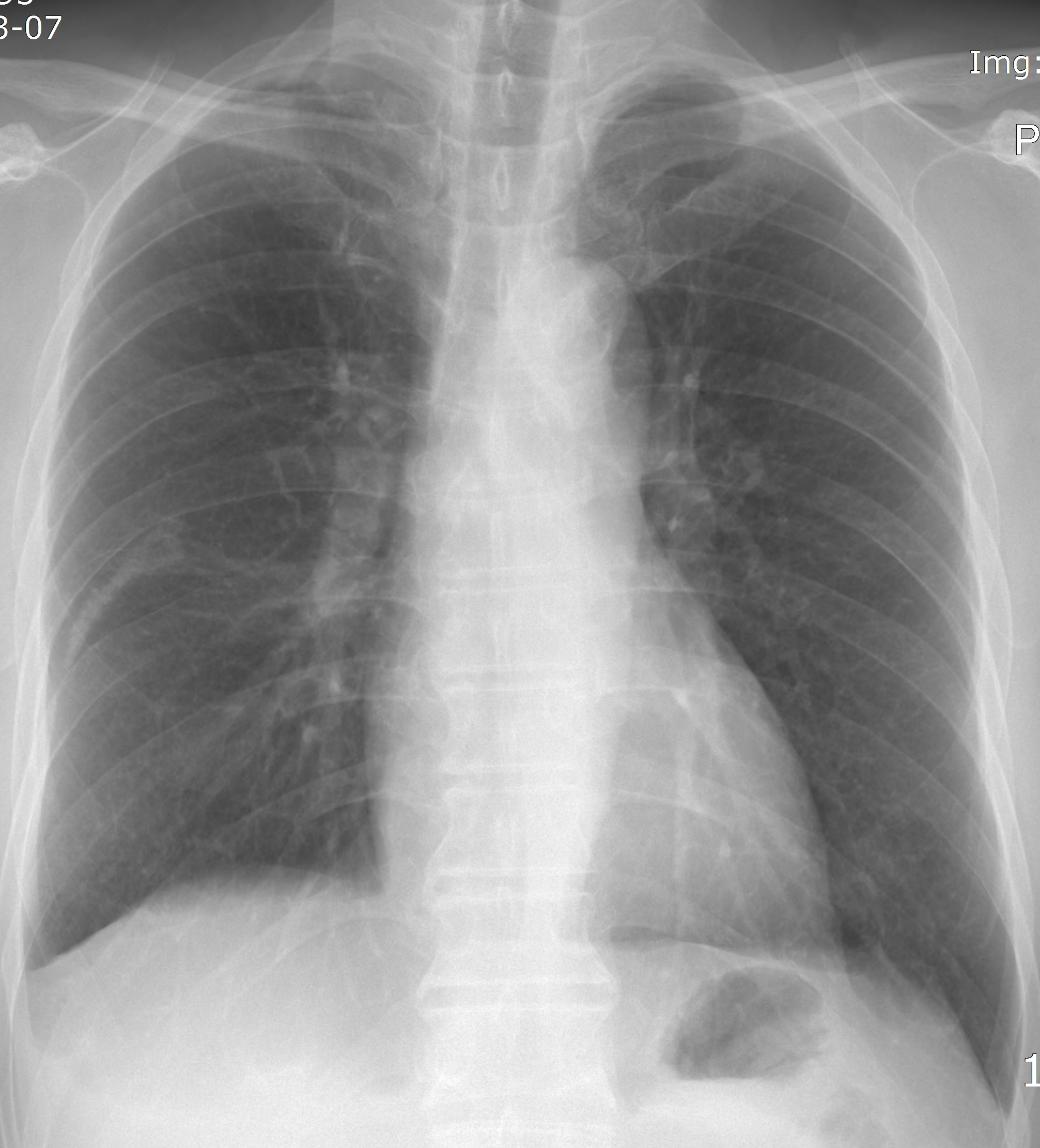

Supplement: S5 Fig — (TIF) [file pone.0164039.s005.tif]

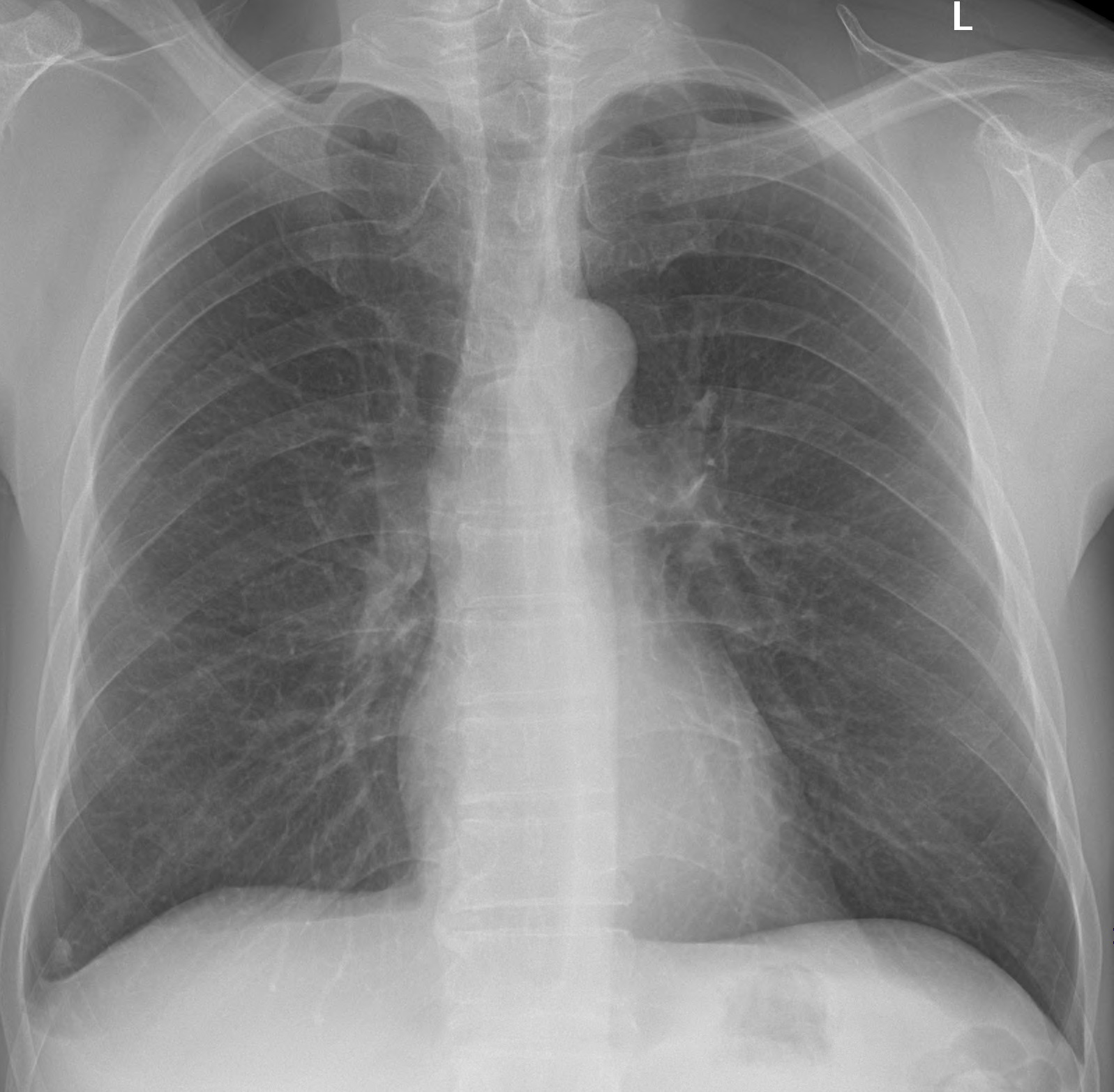

Supplement: S6 Fig — (TIF) [file pone.0164039.s006.tif]

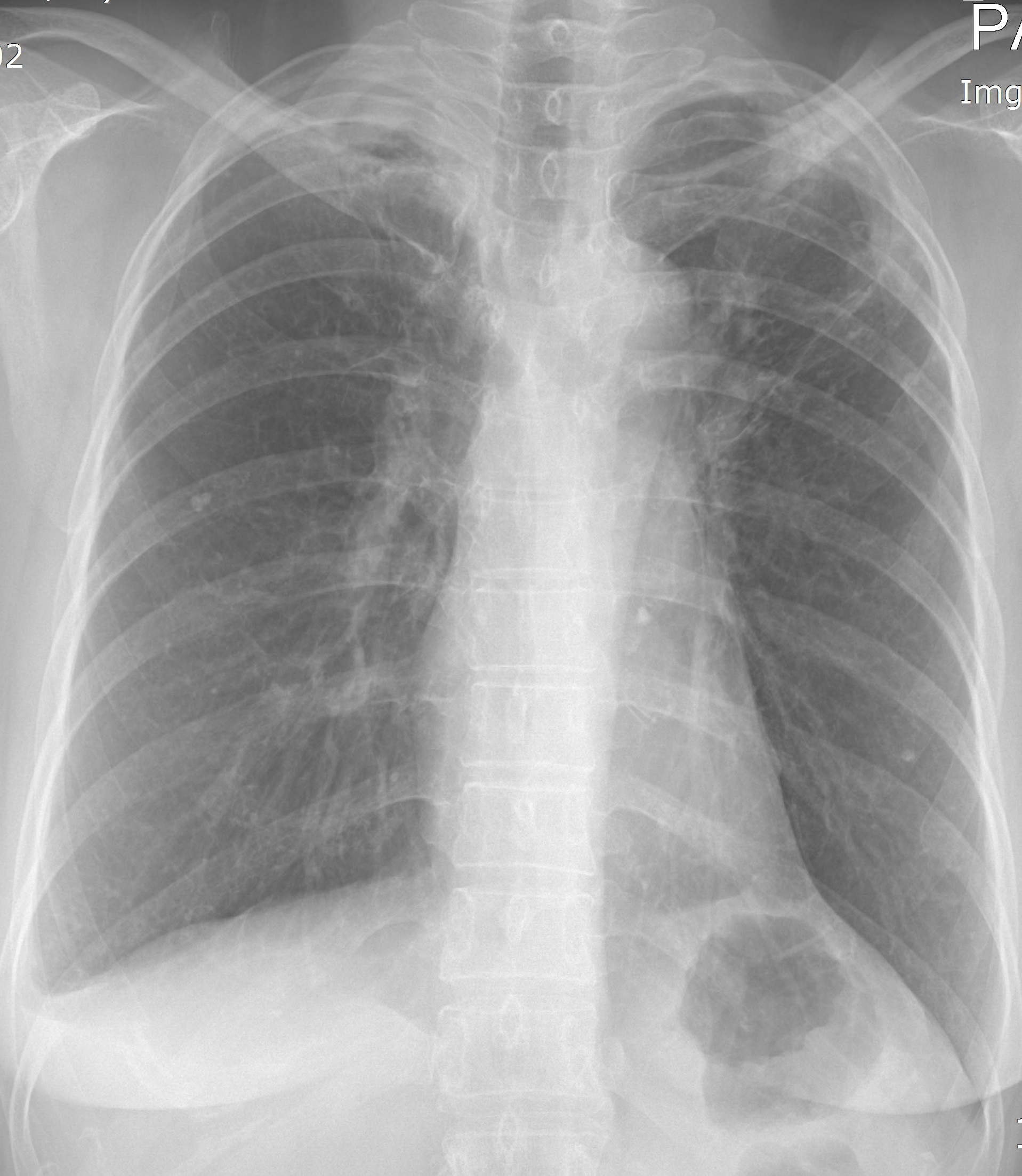

Supplement: S7 Fig — (TIF) [file pone.0164039.s007.tif]
